# PREDICT‐PD: A Two‐Stage Approach to Early Identification of Parkinson's Disease

**DOI:** 10.1002/mdc3.70673

**Published:** 2026-05-18

**Authors:** Sasivimol Virameteekul, Sheena Waters, Brook Huxford, Ashvin Kuri, Manuela Tan, Laura Pérez‐Carbonell, Eduardo de Pablo‐Fernández, Anette Schrag, Alastair J. Noyce, Cristina Simonet

**Affiliations:** ^1^ Centre for Preventive Neurology, Wolfson Institute of Population Health Queen Mary University of London London UK; ^2^ Department of Biochemistry, Faculty of Medicine, Chulalongkorn University and King Chulalongkorn Memorial Hospital Thai Red Cross Society Bangkok Thailand; ^3^ Department of Neurology Oslo University Hospital Oslo Norway; ^4^ Sleep Disorders Centre Guy's and St Thomas’ NHSFoundation Trust London UK; ^5^ Department of Clinical and Movement Neurosciences UCL Institute of Neurology London UK; ^6^ Neurology Department Royal London Hospital, Barts Health NHS Trust London UK

**Keywords:** Parkinson's disease, prodromal Parkinson's disease, early detection, risk stratification, biomarkers

## Introduction

The prodromal phase of Parkinson's disease (PD) represents an important opportunity for both clinical care and research. Isolated REM sleep behavior disorder (iRBD), hyposmia, mood and cognitive complaints, autonomic dysfunction, and subtle motor slowing can precede PD diagnosis by many years, and increasing evidence shows that individuals at substantially elevated risk can be identified.^1^ Early identification enables enrolment of high‐risk individuals into clinical trials, potentially improving detection of disease‐modifying effects, and may also have immediate clinical benefits, including targeted management of neuropsychiatric symptoms and auntonomic dysfunction, as well as sleep disorders, including prevention of sleep‐related injuries, and finally motor dysfunction.

Despite these opportunities, prodromal PD detection remains largely confined to research. Numerous questionnaires, quantitative motor and cognitive tests, and biomarkers have been validated individually, but there is no consensus on how to combine them, in what sequence, or with which thresholds for escalation. Interpretation is further complicated by variability in sensitivity, specificity, and prevalence‐dependent predictive values. Several large longitudinal cohorts have advanced the field over the past decade. Among proposed frameworks, two approaches have been particularly influential. The MDS research criteria for prodromal PD (2015; revised 2019) provide a comprehensive probabilistic model integrating clinical features, imaging, and biomarkers.^2^, ^3^ In parallel, the PREDICT‐PD study has developed a scalable population‐based approach using web‐based questionnaires and remote tasks informed by systematic review and meta‐analysis.^4^, ^5^, ^6^ Recent refinements incorporating likelihood ratios and objective intermediate markers (e.g., tapping speed, hyposmia, RBD symptoms) have improved risk stratification and associations with incident PD and dopamine transporter (DAT) imaging abnormalities.^5^, ^6^ Both MDS criteria and PREDICT‐PD are currently research tools that illustrate complementary strategies: the former emphasizing comprehensiveness, the latter scalability through remote assessment.

Nonetheless, key gaps remain. Although individuals at increased risk can be identified, the sequence and timing of prodromal features, biomarker trajectories, and heterogeneity across subgroups (e.g., iRBD, PAF, hyposmia, or carriers of *GBA1* and *LRRK2* genetic variants) are poorly defined. Emerging biological definitions of PD based on α‐synuclein detection, genetics, and imaging further raise questions regarding alignment with clinical prodromal definitions.^7^, ^8^

In this viewpoint, we propose a two‐phase framework for prodromal PD phenotyping (Fig. 1). Phase 1 uses scalable remote assessments to enrich for at‐risk groups, while Phase 2 involves deep in‐person phenotyping, biomarker integration, and longitudinal follow‐up. To improve protocol scalability, we distinguish core assessments from optional exploratory tasks. Our aim is not to replace existing criteria, but to outline a practical strategy that is feasible for large community studies while remaining informative for clinical and biomarker research.

**Figure 1:**
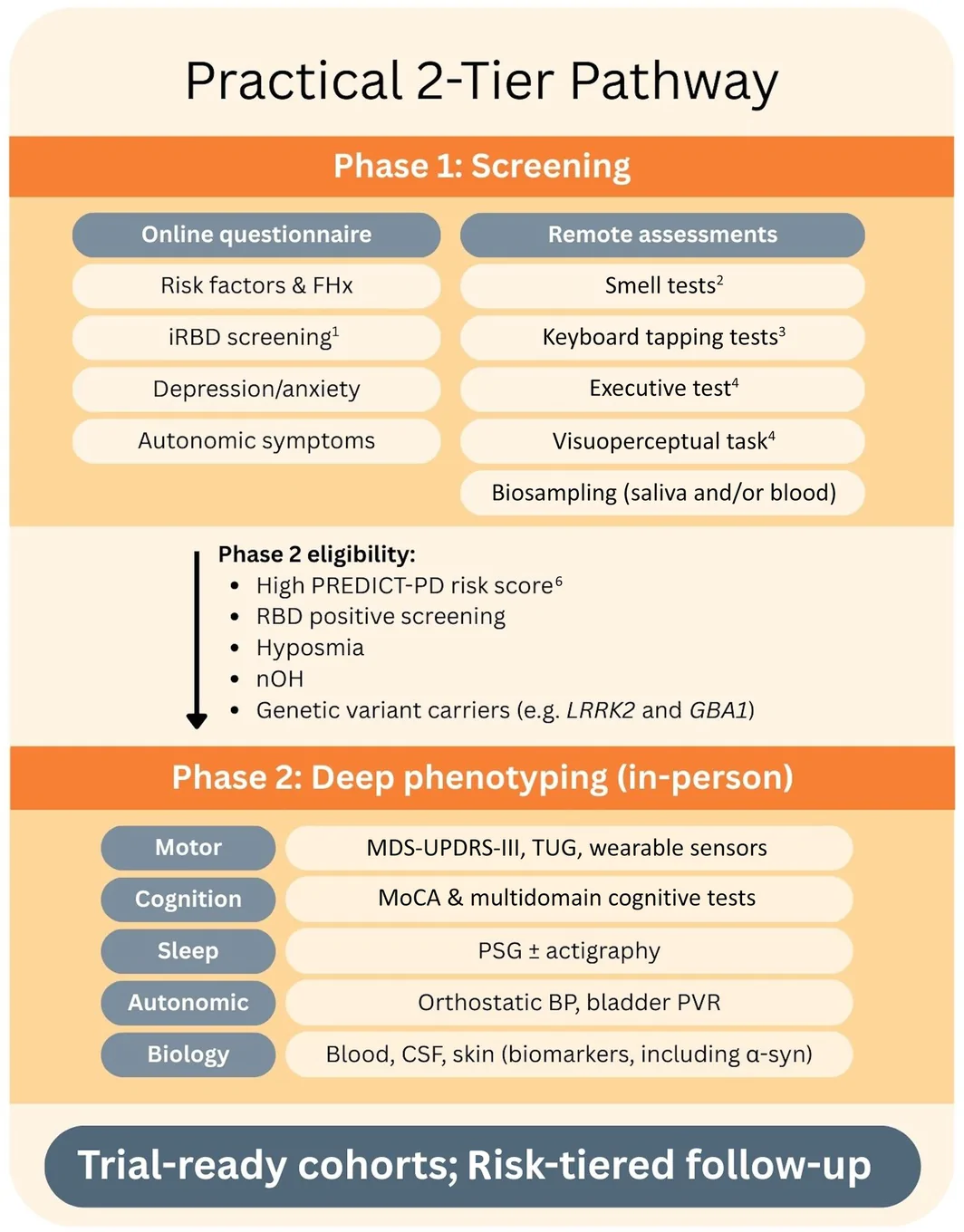
Two‐phase framework for early identification of prodromal Parkinson's disease. A scalable two‐tier pathway using remote screening with targeted escalation to in‐person phenotyping and longitudinal follow‐up. (1) RBD1Q / RBDSQ: Single‐question REM sleep behavior disorder screen/ REM Sleep Behavior Disorder Screening Questionnaire. (2) UPSIT (University of Pennsylvania Smell Identification Test), 6‐item smell test. (3) BRAIN/ DFT tests: Bradykinesia‐Akinesia Incoordination test and Digital Finger Tapping test. (4) TMT: Trail Making Test.  (5) Online Cats‐and‐Dogs test. (6) Risk score based on PREDICT‐PD algorithm. BP, blood pressure; CSF, cerebrospinal fluid; DAT‐SPECT, dopamine transporter single‐photon emission computed tomography; FHx, family history; iRBD: isolated REM sleep behavior disorder; MoCA, Montreal Cognitive Assessment; MRI, magnetic resonance imaging; nOH, neurogenic orthostatic hypotension; PD, Parkinson's disease; PSG, polysomnography; PVR, post‐void residual volume; SAA, seed amplification assay; SN, substantia nigra; TUG, Timed Up‐and‐Go; UPDRS‐III: Unified Parkinson's Disease Rating Scale, motor examination.

## Phase 1: Screening and Risk Stratification

Phase 1 serves as the gateway, designed to identify individuals in the community at increased PD risk. The assessment battery should be concise (≈20–30 min), delivered online with postal smell testing and optional biosampling, allowing periodic re‐administration. Its purpose is enrichment rather than diagnosis, combining demographic risk factors, non‐motor symptoms, intermediate digital markers, and biosampling. Developed and validated through PREDICT‐PD, this approach is acceptable and scalable, with longitudinal assessments demonstrating stable associations between risk scores and intermediate markers over time.^5^, ^6^ For screening efficiency, the default gateway targets adults aged ≥50 years; adults aged 40–49 years may be included when additional enrichment features are present (e.g., first‐degree family history, positive RBD screening, or hyposmia). Age is a central component of the PREDICT‐PD risk algorithm, which begins with an age‐specific baseline probability of PD that is subsequently modified by the presence or absence of additional risk markers.

### Structured Questionnaires and Risk Factor Survey

Participants complete an annual online survey capturing demographics, lifestyle factors, exposures, and medical history. Items include smoking status, coffee and alcohol consumption, pesticide exposure, occupational risks, head injury, and medications. Early non‐motor features, including constipation, urinary dysfunction, erectile dysfunction, anxiety, and depression are recorded alongside family history of PD. Repeated annual assessment enables re‐calculation of risk. In this framework, age, family history of PD (first‐degree relative), iRBD features, hyposmia, and objective motor markers are core enrichment elements; pesticide exposure and head injury are retained as supportive variables in the model rather than stand‐alone escalation triggers.

### Isolated REM Sleep Behavior Disorder

iRBD is screened using the RBD1Q or RBDSQ,^9^, ^10^ with positive screens followed by structured remote interviews. For population screening, RBDSQ is the preferred default tool because it provides graded symptom information; RBD1Q is an acceptable brief alternative when response burden must be minimized. While overnight polysomnography (PSG) is required for diagnosis, high‐yield interview features (e.g., dream enactment behavior with injury) carry strong predictive value. They should prompt Phase 2 referral, consistent with MDS criteria,^2^, ^3^ which weight RBD heavily, but in a format that is scalable to population screening. Questionnaire positivity is treated as an enrichment signal rather than a diagnostic milestone.

### Cognition and Psychological Assessment

Cognitive impairment can occur early in the prodromal phase of PD, particularly within executive domains such as set‐shifting, attention, and processing speed.^3^ The Trail Making Test (TMT) is a simple, scalable tool suitable for community and remote settings. TMT‐A assesses visual search and psychomotor speed, whereas TMT‐B adds task switching and cognitive flexibility. Completion time, error rate, and the TMT‐B/A ratio provide sensitive indices of executive dysfunction. Slower TMT performance has been linked to increased risk of cognitive decline and parkinsonian conversion in at‐risk cohorts. TMT is also recommended by the MDS as part of cognitive evaluation in PD.^11^ In PREDICT‐PD, TMT‐A and TMT‐B are administered using a web‐based version coded and deployed by the study's developers within the online assessment platform, preserving the canonical task structure while enabling automated timing and remote completion.

### Mood and Psychiatric Features

Depression has been associated with a two‐ to threefold increased risk of subsequent PD,^4^ while anxiety and apathy have been linked to faster progression. The Hospital Anxiety and Depression Scale (HADS) is integrated into the annual survey.^12^ Complementary scales such as the Parkinson's Anxiety Scale, or Beck Depression Inventory may be considered in research expansions.^13^ While mood disturbance alone does not mandate escalation, serial measures support calibration of risk models and provide valuable longitudinal data.

### Motor Performance

Motor impairment often emerges late in the prodromal course, but subtle quantitative changes in fine motor control, coordination, and gait can be detected years earlier. Phase 1 therefore uses brief, remote‐friendly metrics, including smartphone‐based tapping tasks and wearable sensors for gait and tremor monitoring. The BRadykinesia Akinesia INcoordination (BRAIN) test is a 30‐s online alternating finger‐tapping task focused on proximal upper limb movements. It provides three variables: Kinesia Score (KS: total taps), Akinesia Time (AT: mean dwell time) and Incoordination Score (IS: variance of time between consecutive keystrokes). A complementary 20‐s distal finger‐tapping (DFT) task assesses distal upper limb movements increasing sensitivity to subtle motor deficits.^14^ Both tests have been validated across PD and iRBD cohorts and are incorporated into the enhanced PREDICT‐PD algorithm as intermediate markers.^15^

### Olfaction

Olfactory impairment is a highly reproducible early feature of PD.^16^ In PREDICT‐PD, postal 40‐item University of Pennsylvania Smell Identification Test (UPSIT‐40) was used in the pilot phase,^17^ while later adoption of 6‐item version to improve adherence and reduce burden.^18^ Odor‐specific likelihood ratios provide greater precision than dichotomised cut‐offs, yet proposed “PD‐specific” odor patterns have not replicated across cohorts, arguing against selective hyposmia unique to PD.^18^ We suggest that hyposmia at screening, defined as performance below the 15th percentile of age‐ and sex‐adjusted normative data or within the lowest tertile^17^, ^19^ should trigger referral to Phase 2.

### Autonomic Features

Constipation, urinary symptoms, orthostatic hypotension, and erectile dysfunction are initially screened using brief single‐item questions. In selected individuals, structured instruments such as SCOPA‐AUT provide multidomain quantification and are validated for remote use.^20^, ^21^, ^22^ Home‐based orthostatic blood pressure testing is feasible and reliable,^23^ and screening for neurogenic orthostatic hypotension (nOH) can be performed using simple heart‐rate/systolic BP ratio criteria,^24^ with abnormal results prompting in‐person autonomic testing in Phase 2.

### Remote Biosampling

Remote saliva collection provides a reliable, high‐yield source of genomic DNA suitable for large‐scale genotyping, enabling integration of polygenic risk scores and targeted variant screening into population‐based prodromal pipelines.^25^, ^26^ This approach has been demonstrated in PREDICT‐PD, where mail‐out saliva kits achieved high return rates and generated data of sufficient quality for genotyping analyses. A similar approach could be expanded to include other remote blood collection devices, enabling blood‐based biomarker analyses, including proteomics and metabolomics, as well as provision of high–molecular weight DNA for genetic studies. Together, remote biosampling supports scalable biobanking and allows longitudinal re‐analysis as new biomarkers emerge.

## Phase 2: Deep Phenotyping of Enriched Groups

Participants would be eligible for Stage 2 enrolment if they meet any of the following: (i) classification as high risk by the PREDICT‐PD algorithm; (ii) a positive RBD1Q or elevated RBDSQ with confirmatory remote interview items; (iii) objective hyposmia on smell testing; (iv) symptomatic nOH based on home‐based orthostatic BP and heart rate measurements; or (v) pathogenic *GBA1* or *LRRK2* variant carriers. These criteria enrich for individuals at increased risk of prodromal synucleinopathy while remaining feasible for community‐based recruitment. Programs may pragmatically prioritize participants in approximately the top 15% higher‐risk stratum for deep phenotyping, depending on local capacity and study aims. Phase 2 does not have to be implemented all at once. Instead, it can be rolled out in parts, depending on resources, capacity, or study priorities. Core elements that can be delivered within general neurology clinics include standardized motor examination (e.g., MDS‐UPDRS‐III), brief multidomain cognitive screening (e.g., MoCA), structured autonomic assessment using validated symptom scales (e.g., SCOPA‐AUT) combined with standardized orthostatic blood pressure measurements, and limited blood sampling. Optional bedside procedures such as post‐void bladder scanning may also be incorporated where available. Additional assessments requiring specialized infrastructure such as wearable‐based motor quantification and overnight video PSG, may be delivered through coordinated regional networks. More resource‐intensive biomarker procedures (e.g., CSF sample, skin biopsy, advanced imaging) would be based in specialist centers.

### Motor Phenotype

The MDS‐UPDRS Part III remains the reference standard for motor assessment,^27^ though its sensitivity in prodromal PD is limited by floor effects, inter‐rater variability, and its design for clinically established disease. In Phase 2, we recommend extending motor evaluation using computer vision analysis of upper and lower limb movements as well as speech and facial recognition through standardized video recordings.

Wearable inertial sensors will enable unsupervised, continuous monitoring of gait (e.g., stride‐time variability, postural sway), hidden tremor and nocturnal mobility features that often precede overt motor signs.^28^ Combining clinical ratings with quantitative metrics is an evidence‐informed strategy for longitudinal benchmarking, but prospective head‐to‐head validation in prodromal cohorts remains needed.^29^, ^30^

### Cognition and Psychological Assessment

In‐person cognitive assessments are used to validate and deepen phenotyping of posterior cortical function. The Montreal Cognitive Assessment (MoCA) provides a practical global screen,^31^ complemented by domain‐specific tests^32^ to capture subtle cognitive changes.

Advanced psychophysical paradigms offer quantitative readouts of early cortical and subcortical dysfunction. Biological‐motion perception tasks, requiring identification of human movement from point‐light displays, reveal impaired visual integration linked to parietal and superior temporal dysfunction and may help to distinguish PD from DLB trajectories.^33^ Object‐invariance tasks, such as the Cats‐and‐Dogs test, detect posterior cortical dysfunction with greater sensitivity than standard visuospatial measures, show minimal ceiling effects, and correlate with MoCA and DLB risk scores. Together, these brief tasks (≈20–25 min) generate scalable continuous metrics that aid modeling and differentiation of prodromal PD from DLB‐leaning trajectories, where visual and social‐perceptual deficits are more prominent.^34^

### Sleep

Overnight PSG is required to confirm iRBD and quantify REM sleep without atonia (RSWA), with supervised laboratory PSG remaining the diagnostic gold standard. Given cost, access, and workforce constraints, laboratory PSG should be prioritized for participants with high pre‐test probability rather than universal use during initial risk screening. Ambulatory PSG and wearable devices may aid screening, but quantitative RSWA burden should be incorporated where feasible given its prognostic value beyond binary classification. Using published assumptions (questionnaire PPV for PSG‐confirmed iRBD of ~15% and annual phenoconversion among confirmed iRBD of ~10%), 100 questionnaire‐positive individuals would be expected to yield approximately 1–2 phenoconverters per year, supporting multi‐marker enrichment.

Beyond iRBD confirmation, integrating subjective and objective sleep measures helps clarify the role of sleep disturbance in neurodegeneration and PD risk. Circadian disruption assessed by actigraphy and obstructive sleep apnoea identified by home sleep testing have both been associated with increased PD risk, while EEG‐based macro‐ and micro‐structural sleep measures from PSG may further inform links between sleep abnormalities and incident PD.

### Autonomic Function

Bedside tests include orthostatic blood pressure and heart rate measurement at 1, 3, and 10 minutes to detect nOH.^35^ Notably, supine orthostatic hypotension is more sensitive and associated with greater symptom burden than seated measures.^36^ Bladder ultrasound for post‐void residual bladder volume (PVR) provides a quick objective measurement of urinary dysfunction; a threshold of >100 mL indicates incomplete emptying, which may be indicative of MSA and >200–300 mL suggests clinically significant retention requiring further evaluation.^37^

### In‐person Biosampling

Phase 2 enables in‐person biosampling to link clinical features with underlying biology. Venepuncture allows collection of blood component for peripheral biomarker analyses and high‐quality DNA, with optional isolation of peripheral blood mononuclear cells (PBMCs) for immune characterization. Where feasible, cerebrospinal fluid (CSF) and skin punch biopsies should be collected for α‐synuclein seed amplification assays (α‐syn SAA) and other neurodegenerative biomarkers, supporting long‐term biobanking, biomarker discovery, and external validation. Within this framework, α‐syn SAA is treated as a biological stratifier rather than a stand‐alone determinant of diagnostic status. SAA positivity may occur in non‐syneucleinopathies,^38^ and SAA‐negative individuals with strong clinical risk profiles remain eligible for longitudinal follow‐up and repeat biological assessment.

### Imaging

Neuroimaging can provide objective confirmation of prodromal neurodegeneration and assist in trajectory stratification, although its use is often center‐specific, not uniformly applied and associated with considerable cost. In PREDICT‐PD, these investigations have not been routinely performed. DAT‐SPECT or dopaminergic PET is most widely used clinically, detecting presynaptic dopaminergic loss, with posterior putaminal reduction predictive of short‐term conversion.^39^ Cardiac MIBG scintigraphy identifies postganglionic sympathetic denervation and helps differentiate Lewy body disorders from MSA. Neuromelanin‐sensitive MRI and quantitative susceptibility mapping remain largely research tools but may detect early substantia nigra and locus coeruleus changes preceding striatal denervation.^40^

### Integrating Multimodal Data and Predictive Modeling

To achieve true trial readiness, multimodal data from phase 2 should be integrated into a unified predictive framework. We propose a three‐level strategy: (i) multivariable models extending PREDICT‐PD–based algorithms by combining clinical, motor, cognitive, autonomic, and biomarker features, using penalized or ensemble methods to reduce overfitting and improve calibration; (ii) longitudinal and survival models (e.g., mixed‐effects, joint longitudinal–survival, time‐to‐event) to estimate both risk and timing of progression while accommodating repeated measures, missingness, and informative dropout; and (iii) multimodal embedding approaches integrating high‐dimensional data (e.g., clinical, molecular and neuroimaging), with representations combined with clinical and demographic features to balance predictive performance and interpretability. Predictive modeling frameworks should be designed to accommodate heterogeneous data availability, allowing core clinical models to function independently, with incremental performance gains using additional biomarker layers.

### Risk Communication and Ethical considerations

Large observational studies (e.g., UK Biobank, PREDICT‐PD) do not necessarily return individual biomarker results. However, there is increasing interest in responsible disclosure, particularly as biological markers such as CSF α‐syn SAA become more predictive. Decisions regarding disclosure should be informed by participatory research and tailored to specific risk groups. Where disclosure is undertaken, risk should be framed as probabilistic and revisable over time, with structured counseling addressing uncertainty and false‐positive risk. A staged approach with opportunities for re‐consent may be appropriate when introducing high‐impact biomarker findings.

## Conclusion

This framework is pragmatic, evidence‐driven, and adaptable across settings. It complements existing research criteria by prioritizing feasibility and while retaining biological depth. Harmonization of protocols and expansion into under‐represented populations reman priorities. As disease‐modifying therapies approach, such a structured approach offers the clearest path to identify, monitor, and support those in the prodromal phase of PD.

## Author Roles

(1) Research project: A. Conception, B. Organization, C. Execution;

(2) Statistical Analysis: Not applicable;

(3) Manuscript Preparation: A. Writing of the first draft, B. Review and Critique.

S.V.: 1A, 1B, 1C, 3A.

S.W.: 1B, 1C, 3B.

B.H.: 1B, 1C, 3B.

A.K.: 1B, 1C, 3B.

M.T.: 1B, 1C, 3B.

L.P.‐C.: 1B, 1C, 3B.

E.d.P.‐F.: 1B, 1C, 3B.

A.S.: 1B, 1C, 3B.

A.N.: 1A, 1B, 3B.

C.S.: 1A, 1B, 3B.

## Disclosures

**Ethical Compliance Statement:** This article is a Viewpoint and did not involve new data collection, patient recruitment, or the analysis of identifiable patient information. Therefore, approval from an institutional review board or ethics committee was not required. Informed patient consent was not required for this work. We confirm that all authors have read the Journal's position on issues involved in ethical publication and affirm that this work is consistent with those guidelines.

**Financial Disclosure and Conflict of Interest:** The authors declare no conflicts of interest related to the research presented in this manuscript. All authors have completed the ICMJE disclosure form. Any additional financial relationships unrelated to the current work during the past year are disclosed therein.

## AUTHOR CONTRIBUTIONS

**Manuela Tan:** Writing – review and editing. **Laura Pérez‐Carbonell:** Writing – review and editing. **Sasivimol Virameteekul:** Conceptualization; methodology; data curation; investigation; formal analysis; writing – review and editing; writing – original draft; visualization; project administration; validation. **Brook Huxford:** Writing – review and editing; visualization. **Anette Schrag:** Writing – review and editing. **Alastair J. Noyce:** Writing – review and editing; conceptualization; project administration; resources; supervision. **Sheena Waters:** Writing – review and editing; validation; software. **Eduardo de Pablo‐Fernández:** Writing – review and editing; supervision. **Cristina Simonet:** Supervision; writing – review and editing; conceptualization; project administration. **Ashvin Kuri:** Writing – review and editing; formal analysis.

## Data Availability

The data that support the findings of this study are available on request from the corresponding author. The data are not publicly available due to privacy or ethical restrictions.

## References

[1] Mahlknecht P , Seppi K , Poewe W . The concept of prodromal Parkinson's disease. J Parkinsons Dis 2015;5(4):681–697. 10.3233/JPD-150685 PMC4927924 26485429

[2] Berg D , Postuma RB , Adler CH , et al. MDS research criteria for prodromal Parkinson's disease. Mov Disord 2015;30(12):1600–1611. 10.1002/mds.26431 26474317

[3] Heinzel S , Berg D , Gasser T , Chen H , Yao C , Postuma RB , MDS Task Force on the Definition of Parkinson's Disease . Update of the MDS research criteria for prodromal Parkinson's disease. Mov Disord 2019;34(10):1464–1470. 10.1002/mds.27802 31412427

[4] Noyce AJ , Bestwick JP , Silveira‐Moriyama L , Hawkes CH , Giovannoni G , Lees AJ , Schrag A . Meta‐analysis of early nonmotor features and risk factors for Parkinson disease. Ann Neurol 2012;72(6):893–901. 10.1002/ana.23687 PMC3556649 23071076

[5] Noyce AJ , R'Bibo L , Peress L , et al. PREDICT‐PD: an online approach to prospectively identify risk indicators of Parkinson's disease. Mov Disord 2017;32(2):219–226. 10.1002/mds.26898 PMC5324558 28090684

[6] Bestwick JP , Auger SD , Simonet C , et al. Improving estimation of Parkinson's disease risk‐the enhanced PREDICT‐PD algorithm. NPJ Parkinsons Dis 2021;7(1):33. 10.1038/s41531-021-00176-9 PMC8017005 33795693

[7] Höglinger GU , Adler CH , Berg D , et al. A biological classification of Parkinson's disease: the SynNeurGe research diagnostic criteria. Lancet Neurol 2024;23(2):191–204. 10.1016/S1474-4422(23)00404-0 38267191

[8] Simuni T , Chahine LM , Poston K , et al. A biological definition of neuronal α‐synuclein disease: towards an integrated staging system for research. Lancet Neurol 2024;23(2):178–190. 10.1016/S1474-4422(23)00405-2 38267190

[9] Postuma RB , Arnulf I , Hogl B , et al. A single‐question screen for rapid eye movement sleep behavior disorder: a multicenter validation study. Mov Disord 2012;27(7):913–916. 10.1002/mds.25037 PMC4043389 22729987

[10] Stiasny‐Kolster K , Mayer G , Schäfer S , Möller JC , Heinzel‐Gutenbrunner M , Oertel WH . The REM sleep behavior disorder screening questionnaire‐‐a new diagnostic instrument. Mov Disord 2007;22(16):2386–2393. 10.1002/mds.21740 17894337

[11] Biundo R , Bezdicek O , Cammisuli DM , et al. Attention/working memory and executive function in Parkinson's disease: review, critique, and recommendations. Mov Disord 2025;40(9):1791–1804. 10.1002/mds.30293 PMC12485586 40678921

[12] Zigmond AS , Snaith RP . The hospital anxiety and depression scale. Acta Psychiatr Scand 1983;67(6):361–370. 10.1111/j.1600-0447.1983.tb09716.x 6880820

[13] Leentjens AFG , Dujardin K , Marsh L , Richard IH , Starkstein SE , Martinez‐Martin P . Anxiety rating scales in Parkinson's disease: a validation study of the Hamilton anxiety rating scale, the Beck anxiety inventory, and the hospital anxiety and depression scale. Mov Disord 2011;26(3):407–415. 10.1002/mds.23184 21384425

[14] Akram N , Li H , Ben‐Joseph A , et al. Developing and assessing a new web‐based tapping test for measuring distal movement in Parkinson's disease: a distal finger tapping test. Sci Rep 2022;12(1):386. 10.1038/s41598-021-03563-7 PMC8748736 35013372

[15] Giovannoni G , van Schalkwyk J , Fritz VU , Lees AJ . Bradykinesia akinesia inco‐ordination test (BRAIN TEST): an objective computerised assessment of upper limb motor function. J Neurol Neurosurg Psychiatry 1999;67(5):624–629. 10.1136/jnnp.67.5.624 PMC1736603 10519869

[16] Doty RL . Olfaction in Parkinson's disease and related disorders. Neurobiol Dis 2012;46(3):527–552. 10.1016/j.nbd.2011.10.026 PMC3429117 22192366

[17] Doty RL , Shaman P , Dann M . Development of the University of Pennsylvania Smell Identification Test: a standardized microencapsulated test of olfactory function. Physiol Behav 1984;32(3):489–502. 10.1016/0031-9384(84)90269-5 6463130

[18] Morley JF , Cohen A , Silveira‐Moriyama L , et al. Optimizing olfactory testing for the diagnosis of Parkinson's disease: item analysis of the university of Pennsylvania smell identification test. NPJ Parkinsons Dis 2018;4:2. 10.1038/s41531-017-0039-8 PMC5768805 29354684

[19] Ponsen MM , Stoffers D , Booij J , van Eck‐Smit BLF , Wolters EC , Berendse HW . Idiopathic hyposmia as a preclinical sign of Parkinson's disease. Ann Neurol 2004;56(2):173–181. 10.1002/ana.20160 15293269

[20] Chaudhuri KR , Schrag A , Weintraub D , Rizos A , Rodriguez‐Blazquez C , Mamikonyan E , Martinez‐Martin P . The movement disorder society nonmotor rating scale: initial validation study. Mov Disord 2020;35(1):116–133. 10.1002/mds.27862 PMC7037759 31571279

[21] Weintraub D , Chaudhuri KR , Schrag A , et al. Validation of the International Parkinson and Movement Disorder Society non‐motor symptoms questionnaire (MDS‐NMS‐Q). Mov Disord 2025;40(6):1037–1046. 10.1002/mds.30202 40251003

[22] Visser M , Marinus J , Stiggelbout AM , Van Hilten JJ . Assessment of autonomic dysfunction in Parkinson's disease: the SCOPA‐AUT. Mov Disord 2004;19(11):1306–1312. 10.1002/mds.20153 15390007

[23] Gibbon JR , Parry SW , Witham MD , Yarnall A , Frith J . Feasibility, reliability and safety of self‐assessed orthostatic blood pressure at home. Age Ageing 2022;51(7):afac153. 10.1093/ageing/afac153 35776671

[24] Kaufmann H , Palma JA . Neurogenic orthostatic hypotension: the very basics. Clin Auton Res 2017;27(Suppl 1):39–43. 10.1007/s10286-017-0437-3 PMC5524853 28620715

[25] Abraham JE , Maranian MJ , Spiteri I , et al. Saliva samples are a viable alternative to blood samples as a source of DNA for high throughput genotyping. BMC Med Genomics 2012;5:19. 10.1186/1755-8794-5-19 PMC3497576 22647440

[26] Nalls MA , McLean CY , Rick J , et al. Diagnosis of Parkinson's disease on the basis of clinical and genetic classification: a population‐based modelling study. Lancet Neurol 2015;14(10):1002–1009. 10.1016/S1474-4422(15)00178-7 PMC4575273 26271532

[27] Goetz CG , Tilley BC , Shaftman SR , et al. Movement Disorder Society‐sponsored revision of the unified Parkinson's disease rating scale (MDS‐UPDRS): scale presentation and clinimetric testing results. Mov Disord 2008;23(15):2129–2170. 10.1002/mds.22340 19025984

[28] Virameteekul S , Shin C , Hirczy SS , et al. Assessing digital health Technologies for Outcome Measurement in Parkinson's disease drug trials: a systematic review. Mov Disord 2026;41(1):40–62. 10.1002/mds.70097 41175008

[29] Sun YM , Wang ZY , Liang YY , Hao CW , Shi CH . Digital biomarkers for precision diagnosis and monitoring in Parkinson's disease. NPJ Digit Med 2024;7(1):218. 10.1038/s41746-024-01217-2 PMC11339454 39169258

[30] Lipsmeier F , Taylor KI , Kilchenmann T , et al. Evaluation of smartphone‐based testing to generate exploratory outcome measures in a phase 1 Parkinson's disease clinical trial. Mov Disord 2018;33(8):1287–1297. 10.1002/mds.27376 PMC6175318 29701258

[31] Nasreddine ZS , Phillips NA , Bédirian V , et al. The Montreal cognitive assessment, MoCA: a brief screening tool for mild cognitive impairment. J Am Geriatr Soc 2005;53(4):695–699. 10.1111/j.1532-5415.2005.53221.x 15817019

[32] Harvey PD . Domains of cognition and their assessment. Dialogues Clin Neurosci 2019;21(3):227–237. 10.31887/DCNS.2019.21.3/pharvey PMC6829170 31749647

[33] Jaywant A , Shiffrar M , Roy S , Cronin‐Golomb A . Impaired perception of biological motion in Parkinson's disease. Neuropsychology 2016;30(6):720–730. 10.1037/neu0000276 PMC5003689 26949927

[34] McKeith IG , Ferman TJ , Thomas AJ , et al. Research criteria for the diagnosis of prodromal dementia with Lewy bodies. Neurology 2020;94(17):743–755. 10.1212/WNL.0000000000009323 PMC7274845 32241955

[35] The Consensus Committee of the American Autonomic Society and the American Academy of Neurology . Consensus statement on the definition of orthostatic hypotension, pure autonomic failure, and multiple system atrophy. Neurology 1996;46(5):1470. 10.1212/wnl.46.5.1470 8628505

[36] Juraschek SP , Appel LJ , C MM , Mukamal KJ , Lipsitz LA , Blackford AL , et al. Comparison of supine and seated orthostatic hypotension assessments and their association with falls and orthostatic symptoms. J Am Geriatr Soc 2022;70(8):2310–2319. 10.1111/jgs.17804 PMC9378443 35451096

[37] Bent AE , Nahhas DE , McLennan MT . Portable ultrasound determination of urinary residual volume. Int Urogynecol J Pelvic Floor Dysfunct 1997;8(4):200–202. 10.1007/BF02765813 9449296

[38] Martinez‐Valbuena I , Fullam S , O'Dowd S , Tartaglia MC , Kovacs GG . Α‐Synuclein seed amplification assay positivity beyond synucleinopathies. EBioMedicine 2025;120:105925. 10.1016/j.ebiom.2025.105925 PMC12489771 40975928

[39] Suwijn SR , van Boheemen CJM , de Haan RJ , Tissingh G , Booij J , de Bie RMA . The diagnostic accuracy of dopamine transporter SPECT imaging to detect nigrostriatal cell loss in patients with Parkinson's disease or clinically uncertain parkinsonism: a systematic review. EJNMMI Res 2015;5(1):12. 10.1186/s13550-015-0087-1 PMC4385258 25853018

[40] Trujillo P , Aumann MA , Claassen DO . Neuromelanin‐sensitive MRI as a promising biomarker of catecholamine function. Brain 2024;147(2):337–351. 10.1093/brain/awad300 PMC10834262 37669320

